# Bacterial Killing Activity of Polymorphonuclear Myeloid-Derived Suppressor Cells Isolated From Tumor-Bearing Dogs

**DOI:** 10.3389/fimmu.2019.02371

**Published:** 2019-10-10

**Authors:** Sabina I. Hlavaty, Yu-Mei Chang, Rachel P. Orth, Mark Goulian, Paul J. Planet, Douglas H. Thamm, Jennifer A. Punt, Oliver A. Garden

**Affiliations:** ^1^Garden Immune Regulation Laboratory, Department of Clinical Sciences and Advanced Medicine, School of Veterinary Medicine, University of Pennsylvania, Philadelphia, PA, United States; ^2^Research Support Office, Royal Veterinary College, London, United Kingdom; ^3^School of Arts and Sciences, University of Pennsylvania, Philadelphia, PA, United States; ^4^Department of Biology, School of Arts and Sciences, University of Pennsylvania, Philadelphia, PA, United States; ^5^Department of Pediatrics, Perelman School of Medicine, University of Pennsylvania, Philadelphia, PA, United States; ^6^Pediatric Infectious Disease Division, Children's Hospital of Philadelphia, Philadelphia, PA, United States; ^7^Flint Animal Cancer Center, Department of Clinical Sciences, Colorado State University, Fort Collins, CO, United States; ^8^Department of Pathobiology, School of Veterinary Medicine, University of Pennsylvania, Philadelphia, PA, United States

**Keywords:** MDSC, PMN-MDSC, G-MDSC, canine, cancer, bactericidal, phagocytosis, reactive oxygen species

## Abstract

Polymorphonuclear myeloid-derived suppressor cells (PMN-MDSCs) are implicated in the progression and outcome of a variety of pathological states, from cancer to infection. Our previous work has identified three antimicrobial peptides differentially expressed by PMN-MDSCs compared to conventional neutrophils isolated from dogs, mice, and human patients with cancer. We therefore hypothesized that PMN-MDSCs in dogs with cancer possess antimicrobial activity. In the current work, we observed that exposure of PMN-MDSCs to Gram-negative bacteria (*Escherichia coli*) increased the expression of reactive oxygen species by the PMN-MDSCs, indicating that they are capable of initiating an anti-microbial response. Electron microscopy revealed that the PMN-MDSCs phagocytosed Gram-negative and Gram-positive (*Staphylococcus aureus*) bacterial species. Lysis of bacteria within some of the PMN-MDSCs suggested bactericidal activity, which was confirmed by the recovery of significantly lower numbers of bacteria of both species following exposure to PMN-MDSCs isolated from tumor-bearing dogs. Our data therefore indicate that PMN-MDSCs isolated from dogs with cancer, in common with PMNs, have phagocytic and bactericidal activity. This nexus of immunosuppressive and antimicrobial activity reveals a hitherto unrecognized function of MDSCs.

## Introduction

Myeloid-derived suppressor cells (MDSCs) are a subset of immunosuppressive myeloid cells that expand under chronic inflammatory conditions. In cancer, MDSCs release reactive oxygen species (ROS) and cytokines such as IL-10, resulting in the suppression of cytotoxic T cells and attenuation of their antineoplastic activity (1, 2). In infections, the immunosuppressive activity of MDSCs may be beneficial or harmful to the host, depending on the context and bacterial targets. In models of pneumonia and *Leishmania major* infection, for example, increased frequencies of MDSCs are associated with improved survival by preventing excessive inflammation (3–5). In contrast, increased frequencies of MDSCs in *Staphylococcus aureus* biofilm infections in a murine model are associated with enhanced T cell suppression and increased bacterial load, reducing survival (6, 7). Recent work has demonstrated the role of the microbiome in driving the expansion of MDSC populations in the context of cancer. A murine model of pancreatic cancer demonstrated an increased bacterial load in the neoplastic pancreas; ablation of the bacterial load by treating wild-type mice with an oral antibiotic regimen attenuated MDSC frequency and improved T cell activation and outcome (8). Such studies therefore suggest a relationship between the ability of MDSCs to respond to microbes and their immunosuppressive activities.

Dogs with naturally occurring cancer are gaining traction as a model to study a variety of biological processes in tumor development. Our work has demonstrated that the polymorphonuclear subset of MDSCs (PMN-MDSCs, also known as granulocytic (G)-MDSCs) isolated from dogs are functionally and phenotypically representative of human PMN-MDSCs, further supporting the dog as a model species. Murine PMN-MDSCs are defined as CD11b^+^Ly6G^+^Ly6C^lo^ peripheral blood mononuclear cells (PBMCs), while human PMN-MDSCs are traditionally defined as CD11b^+^CD14^−^CD15^+^ or CD11b^+^CD14^−^CD66b^+^ PBMCs, with Ly6G, CD15 and CD66b acting as neutrophil (or polymorphonuclear cell; PMN) markers (2). In dogs, we used a parallel marker approach using CADO48A as our canine-specific PMN marker. We found that CD11b^+^CD14^−^CADO48A^+^ PBMCs suppressed T cell function and therefore represented the canine equivalent of PMN-MDSCs (9). Our cross-species transcriptomic analysis revealed that three of the five commonly upregulated genes in PMN-MDSCs isolated from dogs, humans, and mice encode antimicrobial peptides (9). Furthermore, these cells synthesize a number of products attributed to conventional PMN killing of bacteria (2), prompting us to hypothesize that PMN-MDSCs may serve a bactericidal role in certain contexts, including cancer. We show for the first time that PMN-MDSCs isolated from canine cancer patients are able to phagocytose and kill bacteria. Our findings suggest a novel duality of function of MDSCs, raising the possibility that their immunosuppressive function can be modulated by interactions with microbes, which may enhance cancer progression.

## Materials and Methods

### Isolation of Canine Cells

This study was approved by the Institutional Animal Care and Use Committee, and the Privately Owned Animal Protocol Committee (Protocol #500), of the School of Veterinary Medicine, University of Pennsylvania (Penn Vet). Written informed consent was obtained from all owners of dogs sampled in this study. These dogs were patients at the Matthew J Ryan Hospital of Penn Vet. Samples collected at the Flint Animal Cancer Center at Colorado State University were approved under the Clinical Review Board Protocol CS2019-208: Flint Animal Cancer Center Biobanking and Sample Collection. The signalments and clinical diagnoses of the dogs sampled for this study are listed in Supplemental Table 1.

Peripheral blood was aseptically collected from healthy and tumor-bearing dogs, stored at room temperature in the dark, and processed within 24 h. Briefly, blood was diluted 1:1 in sterile Dulbecco's phosphate buffered saline (DPBS) and layered gently over Histopaque-1077 (Sigma-Aldrich, St. Louis, MO, USA). Samples were centrifuged for 30 min at 400 g with acceleration and deceleration set to zero. The PBMC layer was removed using a transfer pipet and transferred to a fresh tube. The remaining serum and Histopaque layer was aspirated and discarded, leaving the red blood cell (RBC) layer. PMNs were isolated from the RBC layer after incubation with 10 times the volume of 1X RBC Lysis Buffer (Multi-Species; Thermo Fisher Scientific, San Diego, CA, USA) for 5 min at room temperature. PBMCs were incubated with RBC Lysis Buffer for 1 min to remove contaminating RBCs. PBMCs and PMNs were then washed with 10% v/v fetal bovine serum (FBS; Hyclone, Logan, UT, USA) in DPBS twice, prior to counting.

PBMCs from healthy control dogs were stained with PE-conjugated anti-dog-CD5 monoclonal antibody (1:200, clone YKIX322.3; Bio-Rad, Hercules, CA, USA). PBMCs from healthy dogs and PBMCs and PMNs from tumor-bearing dogs were stained with PE-Cy7-conjugated anti-dog PMN leukocyte antigen (1:1,600, clone CADO48A; University of Washington, Pullman, WA, USA, https://secure.vetmed.wsu.edu/moab/shop/item.aspx?itemid=246). All staining was performed for 30 min in the dark at 4°C. In our previous publication, we utilized a larger panel to identify canine PMN-MDSCs in a manner that paralleled the panel used to identify human PMN-MDSCs (2, 9). For this study, a simplified, single antigen panel was deployed for FACS^TM^ to conserve reagents and minimize the preparation time of cells prior to setting up bacterial killing assays, following preliminary experiments that demonstrated equivalence of gated cells in the full and abbreviated panels (Supplemental Figure 1). Cells were then washed and resuspended in DPBS containing 2% v/v FBS and 2 mM ethylenediaminetetraacetic acid, and incubated with 4′,6-diamidino-2-phenylindole (DAPI; BioLegend, San Diego, CA, USA) at room temperature in the dark for 10 min, prior to sorting on a BD FACSAria II and analysis on FlowJo® software, version 10.3 (Tree Star, Ashland, OR, USA). PMN-MDSCs were sorted from PBMCs of tumor-bearing dogs, identified as live hypodense CADO48A^+^ granulocytes, while PMNs were sorted from the lysed RBC fraction, identified as live hyperdense CADO48A^+^ granulocytes (healthy control dog: H-PMN, tumor-bearing [cancer] dog: C-PMN). T cells were identified as live CD5^+^ lymphocytes.

### Reactive Oxygen Species Assay

To measure ROS using a modification of a published protocol (10), 5 × 10^5^ cell aliquots of PBMCs and PMNs isolated from four healthy control dogs and six tumor-bearing dogs were loaded with dihydrorhodamine-123 (DHR, Sigma-Aldrich, St. Louis, MO, USA; final concentration = 40 μM) and incubated with or without stimulation at 37°C in a final volume of 200 μl. To induce production of ROS, samples were incubated for 30 min with a 20:1 ratio of *E. coli* to cells. To inhibit ROS production, diphenyleneiodonium (DPI, Sigma-Aldrich) was added to a final concentration of 19.1 μM. After incubation, samples were immediately placed on ice and washed in 1 mL of cold PBS. PBMCs were subsequently resuspended in 100 μL of cold PBS, stained with 0.5 μg anti-CADO48A [conjugated with either APC (Bio-Rad) or PE-Cy7 (Bio-Rad)], incubated on ice in the dark for 30 min, then washed with 1 mL of cold PBS. Stained PMNs and PBMCs were resuspended in 350 μL of staining medium (PBS; 0.1%BSA; 0.1%NaN_3_) for analysis via flow cytometry on a FACSCalibur^TM^ and analyzed using FlowJo^®^ software, version 10.6.

### Bacterial Killing Assay

Our bacterial killing assay was modified from a published protocol (11). Single colonies of *E. coli* (strain MG1655) and *S. aureus* (RN6607; strain 502A) were grown as an overnight culture, diluted the next morning 1:10 in sterile Luria-Bertani (LB) broth, and grown at 250 rotations per minute (rpm) to an optical density at 600 nm of 1.0, before placing on ice. Prior to incubation with canine cells (*E. coli*: eleven healthy control dogs, six tumor-bearing dogs; *S. aureus*: eight healthy control dogs, five tumor-bearing dogs), bacteria were diluted 1:10 in DPBS and grown for 30 min at 80 rpm at 37°C, before resuspension in Roswell-Park Memorial Institute (RPMI)-1640 medium (Life Technologies, Carlsbad, CA, USA) containing 10 mM HEPES. Canine cells (2 × 10^5^ cells in 50 μL) were incubated for 15 min alone at room temperature in a round bottom 96-well plate, after which the bacteria were added at a ratio of bacteria: cells of 10:1. The plate was centrifuged at 500 g for 5 min, before incubation at 37°C for 40 min at 80 rpm. Serial dilutions of each condition were prepared in 0.1% Triton-X in sterile water in order to release any viable, internalized bacteria by lysis, before the preparation of LB plates that were incubated overnight to count resulting colony-forming units (CFUs) the next day. Co-culture CFUs were normalized to CFUs for bacteria alone.

### Electron Microscopy

Canine cells were isolated from one tumor-bearing dog and one healthy control dog, and incubated with *E. coli or S. aureus* as described above. After centrifugation at 500 g for 10 min, the cells were resuspended in 1 mL of fixative buffer (2.5% glutaraldehyde, 2.0% paraformaldehyde in 0.1 M sodium cacodylate buffer, pH 7.4) for 30 min at room temperature. After storage at 4°C for up to 16 h, the cells were washed with 0.1 M sodium cacodylate at pH 7 and post-fixed in 2.0% osmium tetroxide for 1 h at room temperature, before another wash in buffer and then distilled water. After dehydration through a graded ethanol series, the cells were embedded in Embed-812 (Electron Microscopy Sciences, Fort Washington, PA). Thin sections were stained with uranyl acetate and lead citrate, before examination with a JEOL 1010 electron microscope fitted with a Hamamatsu digital camera and AMT Advantage image capture software.

Approximately 100 images of each cell type were collected in a grid-like and unbiased manner for quantification. All images for quantification were collected at a magnification of 15,000 × . The images were scrambled using random.org, before review of all images in a blinded manner to assess the number of bacteria internalized, and endoplasmic reticulum (ER) dilation score. At least half of the cross-sectional profile of a bacterium had to be internalized by the canine cell to be counted as internal. Endoplasmic reticulum dilation score was determined as previously published (12): dilated ER not observed in the cytoplasm (score 0), dilated ER present in up to one third of the cytoplasm (score 1), one third to two thirds of the cytoplasm (score 2), or more than two thirds of the cytoplasm (score 3).

### Statistics

Linear mixed effects models were used to evaluate differences in normalized percentage DHR positivity and normalized median fluorescence intensity (MFI) between conditions, cell types and their interactions, in which subject dog identification was included as a random effect. Both DHR percentage and MFI were skewed, prompting log transformation prior to analysis. Poisson regression and ordinal logistic regression were used to compare bacterial count or dilated ER score between cell types. For *E. coli* and *S. aureus* killing assays, linear mixed effects models were adopted to compare cell types and bacteria; experimental date and dog were considered as random effects. Raw frequency was log-transformed prior to analysis. Fisher's Least Significant Difference was adopted for all *post-hoc* comparisons.Frequencies are displayed as mean ± standard deviation (SD) or median [inter-quartile range (IQR)], as appropriate. All analyses were carried out in R, version 3.5.1 (R Foundation for Statistical Computing; Vienna, Austria).

## Results

### Bacteria Elicit the Synthesis of Reactive Oxygen Species by PMN-MDSCs

**Figure 1:**
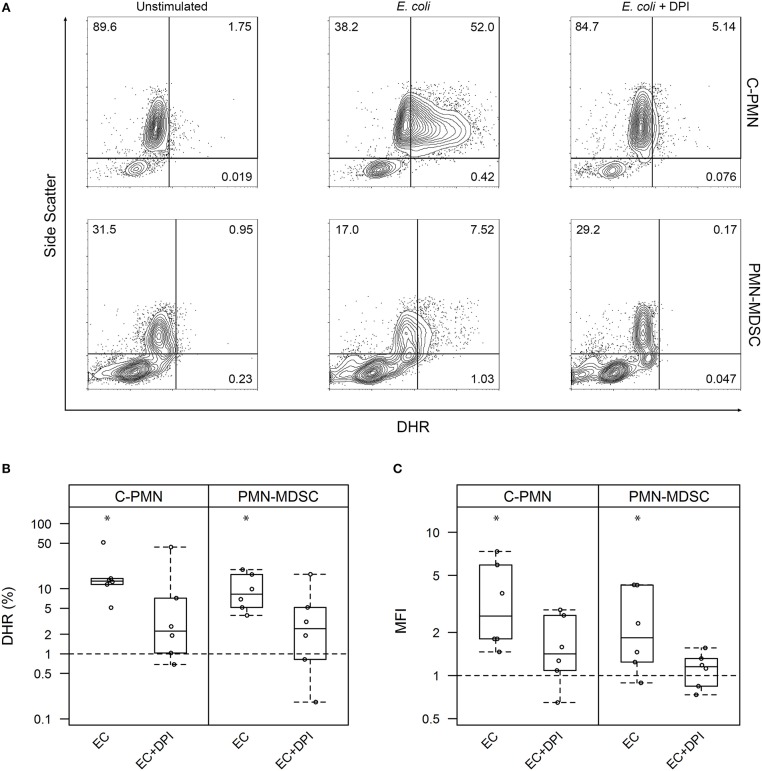
Exposure of PMNs and PMN-MDSCs from tumor-bearing dogs to *E. coli* leads to production of reactive oxygen species. **(A)** Exemplar of flow cytometric data for C-PMNs and PMN-MDSCs isolated from the same tumor-bearing dog. Numbers indicate frequency of total cells that fall within each gate. **(B,C)** Summary of all experiments displaying results as a measure of **(B)** proportion of CADO48A^+^ cells that are DHR^+^ and **(C)** MFI of CADO48A^+^ cells on the DHR channel. DPI was used to inhibit NADPH oxidase production of ROS. Percentage or MFI of DHR^+^ cells following exposure to *E. coli* is normalized to the percentage or MFI of DHR^+^ cells under the unstimulated condition. An asterisk denotes *p* < 0.01 and is based on a least square means analysis, comparing each condition to the normalized unstimulated condition (*y* = 1). Each dot represents a different dog. Box-and-whisker plots display the 25th and 75th percentile with median indicated by the center line, while the whiskers indicate the lowest and the highest data points still within 1.5 times the interquartile range of the respective lower and upper quartiles. C, cancer; PMN, polymorphonuclear cell; ROS, reactive oxygen species; MFI, mean fluorescence intensity; DPI, diphenyleneiodonium; DHR, dihydrorhodamine-123; EC, *E. coli*.

Given that PMN-MDSC suppressive activity is attributed partially to their production of ROS (1), and ROS mediate bacterial killing (13), we first set out to ask whether exposure of PMN-MDSCs to bacteria increased cellular ROS synthesis. We loaded canine cells with DHR and measured its oxidation by ROS, which results in a green fluorescent product that can be detected by flow cytometry (Figure 1A). Exposure of both C-PMNs (*p* = 0.0012) and PMN-MDSCs (*p* = 0.0062) to *E. coli* increased the percentage of DHR^+^ CADO48A^+^ cells when compared to canine cells alone, indicating an increase in ROS production. This phenomenon was extinguished when NADPH oxidase was inhibited with DPI (C-PMN: *p* = 0.122, PMN-MDSC: *p* = 0.33; Figure 1B). Comparison of the MFI for each condition yielded similar observations. *E. coli* once again elicited an increased DHR MFI (C-PMN: *p* = 0.00012, PMN-MDSC: *p* = 0.0086), which was inhibited by DPI (C-PMN: *p* = 0.13, PMN-MDSC: *p* = 0.74; Figure 1C). PMN-MDSCs therefore produce ROS in an NADPH-dependent manner in direct response to bacteria.

### PMN-MDSCs Phagocytose *E. coli* and *S. aureus*

**Figure 2:**
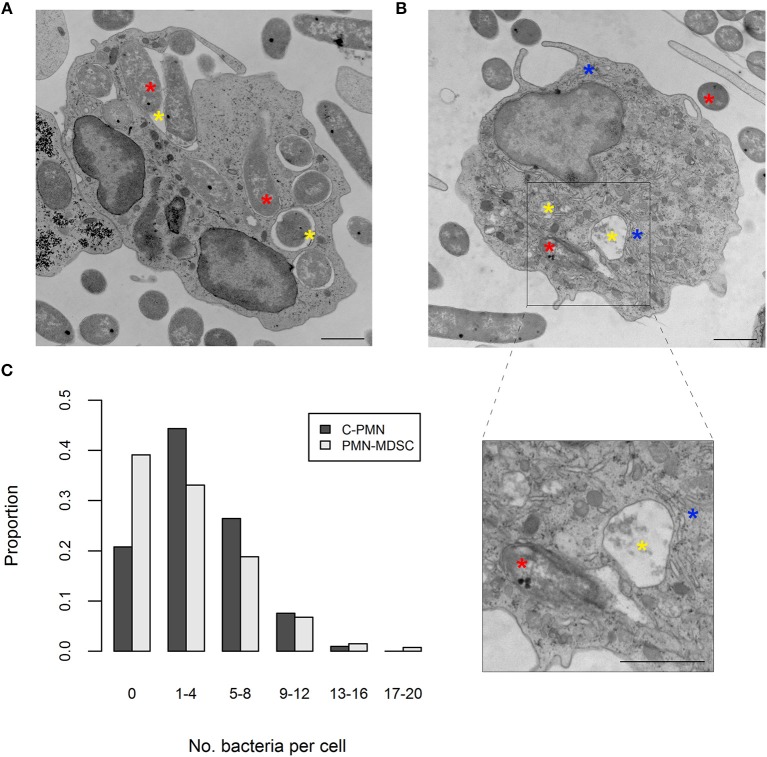
PMN-MDSCs phagocytose bacteria. **(A,B)** EM images of **(A)** C-PMN and **(B)** PMN-MDSC isolated from tumor-bearing dogs. Blue asterisks indicate dilated endoplasmic reticulum, yellow asterisks indicate phagolysosomes, and red asterisks indicate *E. coli*. Scale bar = 1 μm. **(C)** Bar graph depicting the proportion of total cells of each cell type analyzed by EM that had the respective range of bacteria internalized. C, cancer; PMN, polymorphonuclear cell; EM, electron microscopy.

ROS production was enhanced in PMN-MDSCs exposed to bacteria in an NADPH-oxidase-dependent manner. This phenomenon is known to accompany phagocytosis (13), prompting us to ask whether PMN-MDSCs are phagocytic. While T cells did not phagocytose *E. coli* (a negative control in these assays; data not shown), PMN-MDSCs showed clear evidence of phagocytosis, in common with C-PMNs (Figures 2A,B). Identity of the PMN-MDSCs was verified by analysis of dilated ER (Supplemental Figure 2A) (9, 12). Both populations had a similar range of internalized *E. coli* present in the cytoplasm per cell (C-PMNs: 0–16, PMN-MDSCs: 0–19; Figure 2C, Supplemental Figure 2B), although median [IQR] numbers of bacteria per cell were marginally lower in PMN-MDSCs (1 [5]) compared to C-PMNs (3 [5]; *p* = 0.0044). PMN-MDSCs also showed evidence of phagocytosis of *S. aureus* (Supplemental Figure 3). PMN-MDSCs are therefore able to phagocytose both Gram-negative and Gram-positive bacteria.

### PMN-MDSCs Exhibit Bactericidal Activity

**Figure 3:**
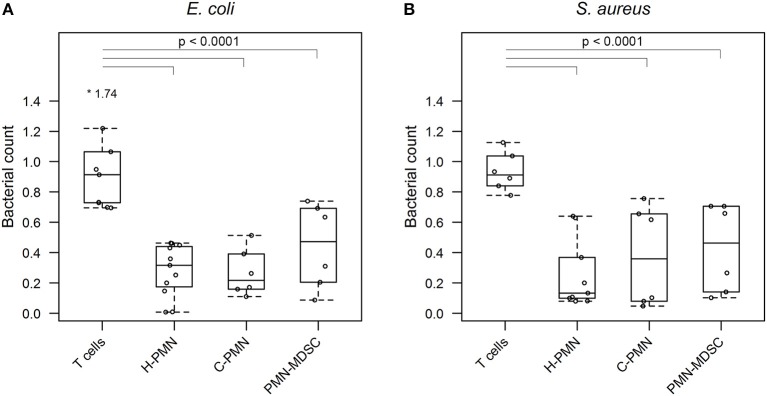
PMN-MDSCs exhibit bactericidal activity. Summary data from bacterial killing assays co-incubating **(A)** *E. coli* or **(B)** *S. aureus* with T cells and PMNs isolated from healthy dogs, and PMNs and PMN-MDSCs isolated from tumor-bearing dogs. Bacterial growth with canine cells was normalized to growth of bacteria alone. A linear mixed effects approach was used for statistical analyses, with the statistically significant comparisons indicated by a solid black line. Each dot represents a different dog. Boxes indicate 25^th^ and 75^th^ percentile with median graphed in the center, while whiskers indicate the lowest and highest data points within 1.5 times the interquartile range of the upper and lower limits. Outlier results are indicated with an asterisk. H, healthy; C, cancer; PMN, polymorphonuclear cell.

Having confirmed that PMN-MDSCs are able to phagocytose *E. coli*, we next asked whether PMN-MDSCs kill bacteria. The growth of bacteria exposed to PMN-MDSCs was significantly lower, when normalized to bacteria alone, than a negative control population of T cells (PMN-MDSCs: 0.445 ± 0.278, T cells: 0.971 ± 0.340, *p* = 2.6 × 10^−8^; Figure 3A). Similarly, PMNs isolated from both healthy control dogs (0.282 ± 0.172; *p* < 2 × 10^−16^) and tumor-bearing dogs (0.268 ± 0.156; *p* = 1.5 × 10^−13^) inhibited bacterial growth. PMN-MDSCs (0.430 ± 0.291) also showed enhanced bactericidal activity against *S. aureus* compared to T cells (0.934 ± 0.128, *p* = 1.5 × 10^−7^; Figure 3B). Similar results were observed for PMNs isolated from healthy control (0.260 ± 0.231, *p* = 6.7 × 10^−12^) and tumor-bearing (0.376 ± 0.332, *p* = 9.0 × 10^−9^) dogs. PMN-MDSCs isolated from tumor-bearing dogs are therefore able to kill both Gram-negative and Gram-positive bacteria.

## Discussion

PMN-MDSCs promote an immunosuppressive microenvironment, which may be beneficial or harmful to the host depending on circumstances (14). In the context of cancer, they play an important role in suppressing T cell activity and promoting tumor development (1, 2). However, many questions about PMN-MDSC function remain unanswered, including the possibility that they serve roles other than suppression in certain contexts. Capitalizing on our former studies of canine MDSCs and previous work suggesting that MDSCs may be phagocytic in certain contexts (4, 9), we set out to address whether PMN-MDSCs isolated from tumor-bearing dogs have bacterial killing activity.

Since production of ROS as part of the oxidative burst has been linked to killing of bacteria by PMNs (13), and PMN-MDSCs utilize ROS as one of the mechanisms of suppression, we first asked whether exposure to bacteria elicited ROS production in canine PMN-MDSCs. We found that exposure to *E. coli* increased the concentration of ROS in PMN-MDSCs in an NADPH oxidase-dependent manner, suggesting that *E. coli* interactions with PMN-MDSCs stimulate downstream signaling pathways that culminate in ROS production.

We next wished to understand whether PMN-MDSCs from tumor-bearing dogs are able to phagocytose bacteria. This aspect of PMN-MDSC function has not been studied as extensively as it has in PMNs; however, a number of studies in a variety of contexts have found these cells to be capable of phagocytic activity. PMN-MDSCs isolated from tumor-bearing mice were able to phagocytose latex beads (15), while PMN-MDSCs isolated from infected mice phagocytosed Gram-negative bacteria, although not as proficiently as PMNs (16). Similarly, PMN-MDSCs isolated from human cord blood phagocytosed both Gram-positive and Gram-negative bacteria (4). However, to the best of our knowledge the phagocytosis of living Gram-positive and Gram-negative bacteria by PMN-MDSCs has not been investigated in the context of cancer. Confirming by electron microscopy that PMN-MDSCs isolated from dogs with cancer are able to phagocytose both *E. coli* and *S. aureus*, we extended these observations by demonstrating that PMN-MDSCs have a direct bactericidal function. This phenomenon was consistent with our observation of bacterial debris in phagolysosomes within some of the PMN-MDSCs we imaged. Interestingly, the median number of bacteria per cell was higher in C-PMNs than in PMN-MDSCs, but the difference was marginal and of questionable biological significance. While these results may indicate that PMN-MDSCs are intrinsically less phagocytic than PMNs, several variables—such as random plane of section, the limitations of static images, the limited number of dogs used for imaging, and our interrogation of only two bacterial species—precluded reliable quantitative comparisons of phagocytic efficiency in our experiments. Further work will be required to address the comparative phagocytic ability of PMN-MDSCs and PMNs.

In summary, our findings highlight a novel bacterial killing function of PMN-MDSCs isolated from tumor-bearing dogs. This adds another function to PMN-MDSCs' repertoire of activities and raises intriguing questions about how PMN-MDSCs might be involved in establishing a pre-neoplastic niche in tumors associated with certain bacteria (17–19). For example, we speculate that PMN-MDSCs function in regions of bacterial colonization or infection in order to target the bacteria, yet in doing so promote an immune suppressive microenvironment that drives aggressive expansion of neoplastic cells (20–22). We hypothesize that PMN-MDSCs promote a suppressive microenvironment early in certain bacterial infections, contributing to the development of a pre-neoplastic niche and tumor development. The nexus of suppressive and bactericidal MDSC function may therefore represent an important focus of future research into oncogenesis.

## Data Availability Statement

The datasets generated for this study are available on request to the corresponding author.

## Ethics Statement

The animal study was reviewed and approved by University of Pennsylvania's Institutional Animal Care and Use Committee, the Privately Owned Animal Protocol Committee (Protocol #500) at the School of Veterinary Medicine, University of Pennsylvania, and the Clinical Review Board Protocol CS2019-208: Flint Animal Cancer Center Biobanking and Sample Collection at the Flint Animal Cancer Center at Colorado State University. Written informed consent was obtained from the owners for the participation of their animals in this study.

## Author Contributions

SH and OG conceived and planned the experiments. SH processed samples for EM, collected and analyzed EM images, performed bacterial killing assays, and wrote the first draft of the manuscript. Y-MC performed statistical analyses and created summary figures. RO performed the oxidative burst assays under the guidance of JP. DT provided samples from tumor-bearing dogs. PP and MG provided bacterial strains and advice on bacterial assays. OG funded the project, supervised SH, and edited all drafts of the manuscript. All authors read and approved the final draft of the manuscript.

### Conflict of Interest

The authors declare that the research was conducted in the absence of any commercial or financial relationships that could be construed as a potential conflict of interest.
