# Supplementary material for: Bacterial Killing Activity of Polymorphonuclear Myeloid-Derived Suppressor Cells Isolated From Tumor-Bearing Dogs
Source: Front Immunol. 2019 Oct 10;10:2371. doi: 10.3389/fimmu.2019.02371 (PMC6795752; doi:10.3389/fimmu.2019.02371)
Supplement: Supplementary file 1 [file Presentation_1.pptx]

## Slide 1
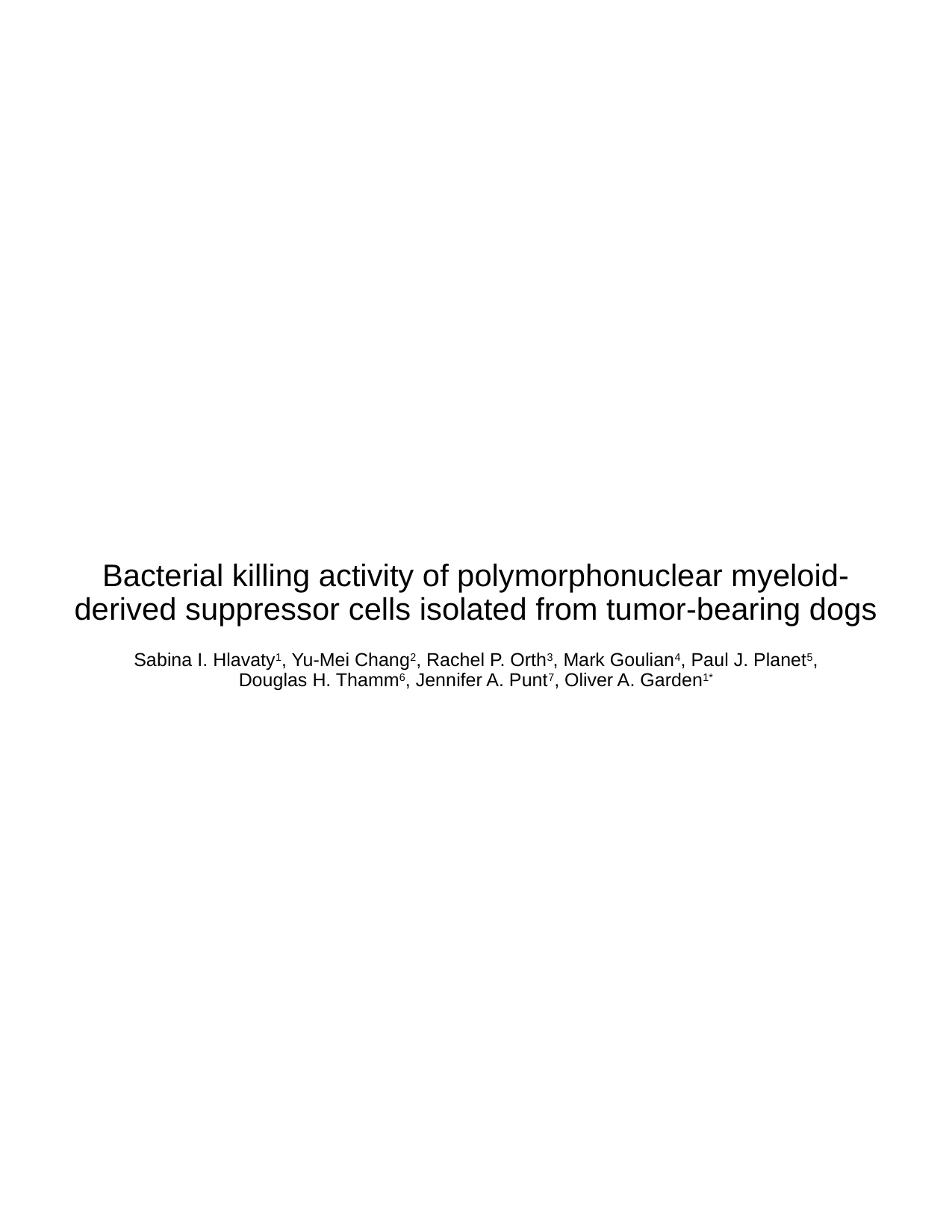

# Bacterial killing activity of polymorphonuclear myeloid-derived suppressor cells isolated from tumor-bearing dogs
Sabina I. Hlavaty1, Yu-Mei Chang2, Rachel P. Orth3, Mark Goulian4, Paul J. Planet5, Douglas H. Thamm6, Jennifer A. Punt7, Oliver A. Garden1*

## Slide 2
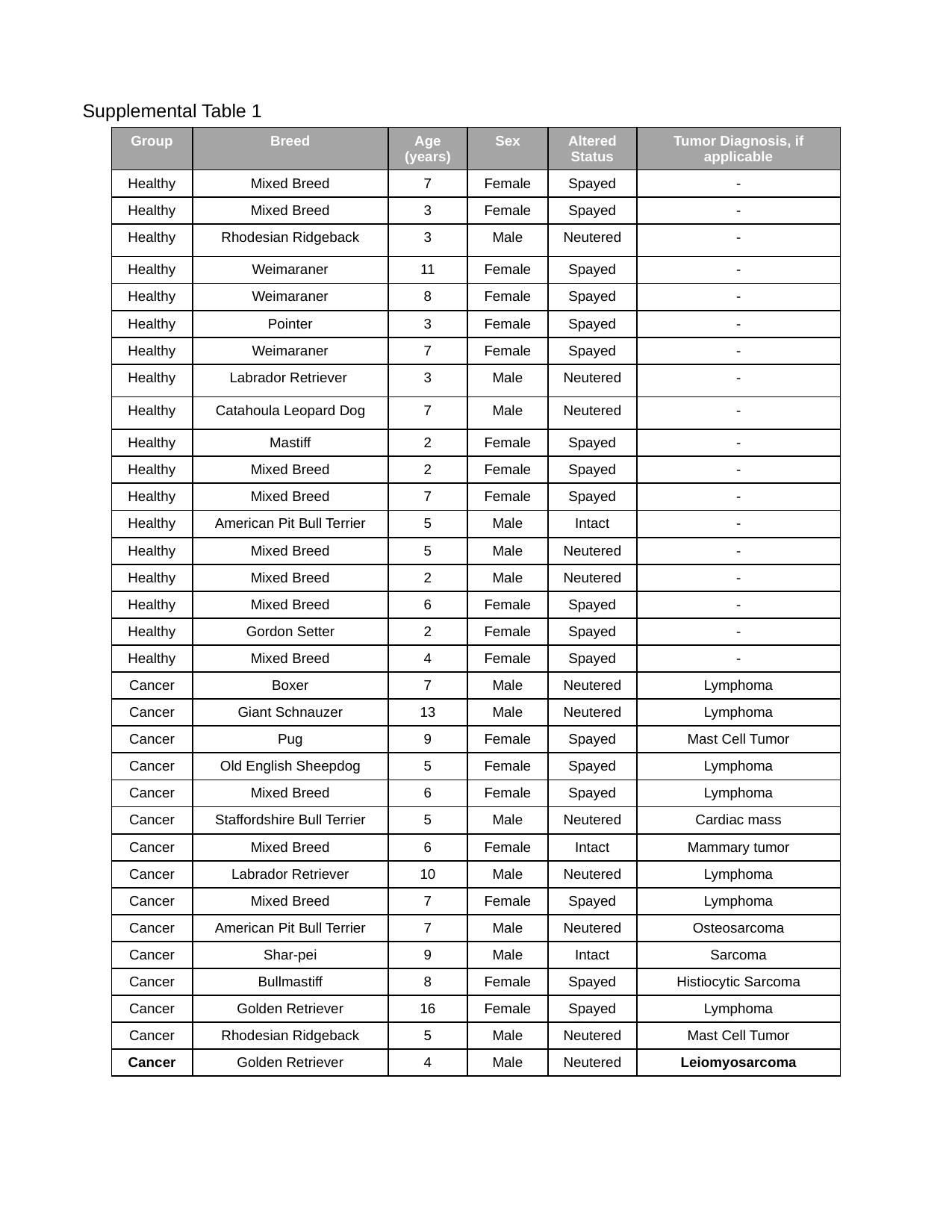

Supplemental Table 1
| Group | Breed | Age (years) | Sex | Altered Status | Tumor Diagnosis, if applicable |
| --- | --- | --- | --- | --- | --- |
| Healthy | Mixed Breed | 7 | Female | Spayed | - |
| Healthy | Mixed Breed | 3 | Female | Spayed | - |
| Healthy | Rhodesian Ridgeback | 3 | Male | Neutered | - |
| Healthy | Weimaraner | 11 | Female | Spayed | - |
| Healthy | Weimaraner | 8 | Female | Spayed | - |
| Healthy | Pointer | 3 | Female | Spayed | - |
| Healthy | Weimaraner | 7 | Female | Spayed | - |
| Healthy | Labrador Retriever | 3 | Male | Neutered | - |
| Healthy | Catahoula Leopard Dog | 7 | Male | Neutered | - |
| Healthy | Mastiff | 2 | Female | Spayed | - |
| Healthy | Mixed Breed | 2 | Female | Spayed | - |
| Healthy | Mixed Breed | 7 | Female | Spayed | - |
| Healthy | American Pit Bull Terrier | 5 | Male | Intact | - |
| Healthy | Mixed Breed | 5 | Male | Neutered | - |
| Healthy | Mixed Breed | 2 | Male | Neutered | - |
| Healthy | Mixed Breed | 6 | Female | Spayed | - |
| Healthy | Gordon Setter | 2 | Female | Spayed | - |
| Healthy | Mixed Breed | 4 | Female | Spayed | - |
| Cancer | Boxer | 7 | Male | Neutered | Lymphoma |
| Cancer | Giant Schnauzer | 13 | Male | Neutered | Lymphoma |
| Cancer | Pug | 9 | Female | Spayed | Mast Cell Tumor |
| Cancer | Old English Sheepdog | 5 | Female | Spayed | Lymphoma |
| Cancer | Mixed Breed | 6 | Female | Spayed | Lymphoma |
| Cancer | Staffordshire Bull Terrier | 5 | Male | Neutered | Cardiac mass |
| Cancer | Mixed Breed | 6 | Female | Intact | Mammary tumor |
| Cancer | Labrador Retriever | 10 | Male | Neutered | Lymphoma |
| Cancer | Mixed Breed | 7 | Female | Spayed | Lymphoma |
| Cancer | American Pit Bull Terrier | 7 | Male | Neutered | Osteosarcoma |
| Cancer | Shar-pei | 9 | Male | Intact | Sarcoma |
| Cancer | Bullmastiff | 8 | Female | Spayed | Histiocytic Sarcoma |
| Cancer | Golden Retriever | 16 | Female | Spayed | Lymphoma |
| Cancer | Rhodesian Ridgeback | 5 | Male | Neutered | Mast Cell Tumor |
| Cancer | Golden Retriever | 4 | Male | Neutered | Leiomyosarcoma |

## Slide 3
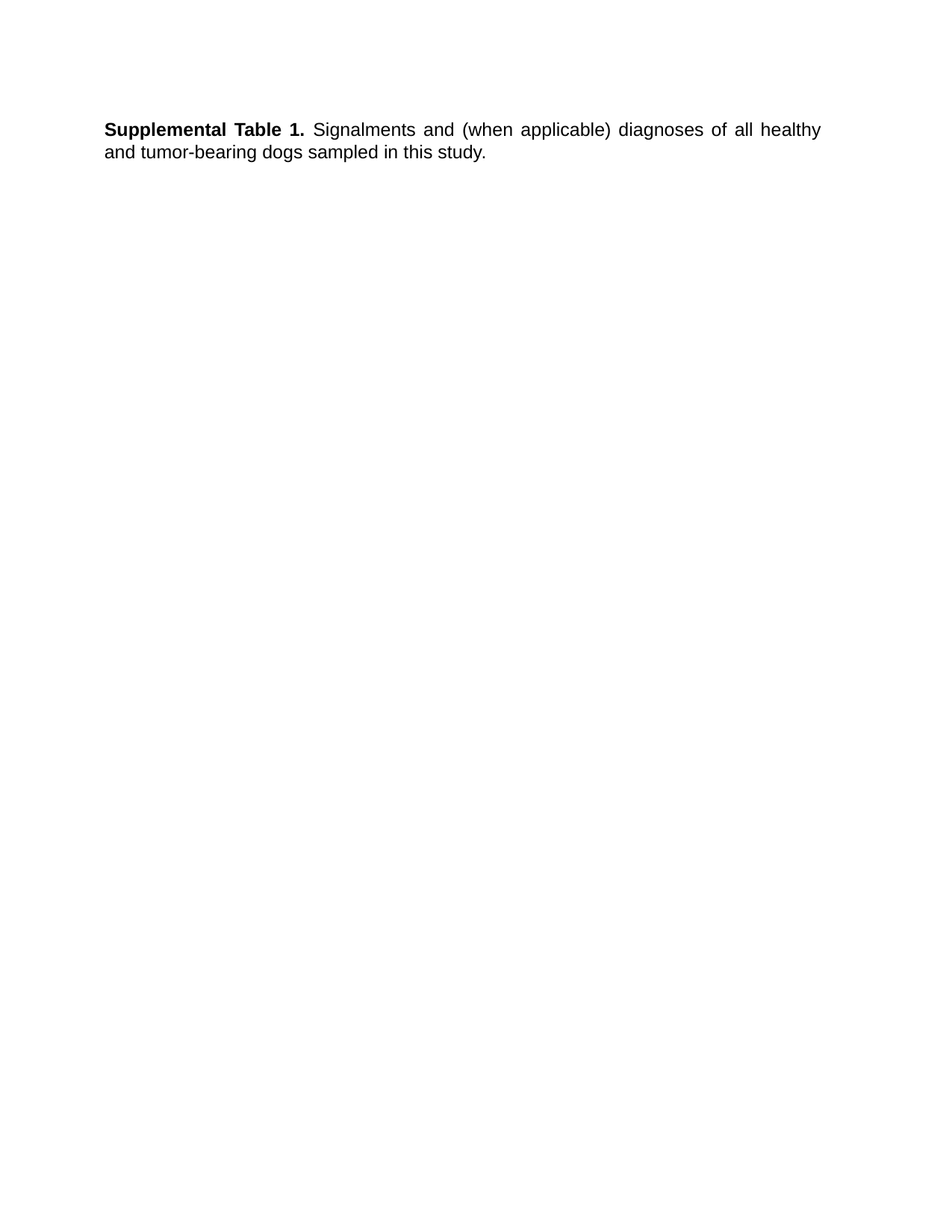

Supplemental Table 1. Signalments and (when applicable) diagnoses of all healthy and tumor-bearing dogs sampled in this study.

## Slide 4
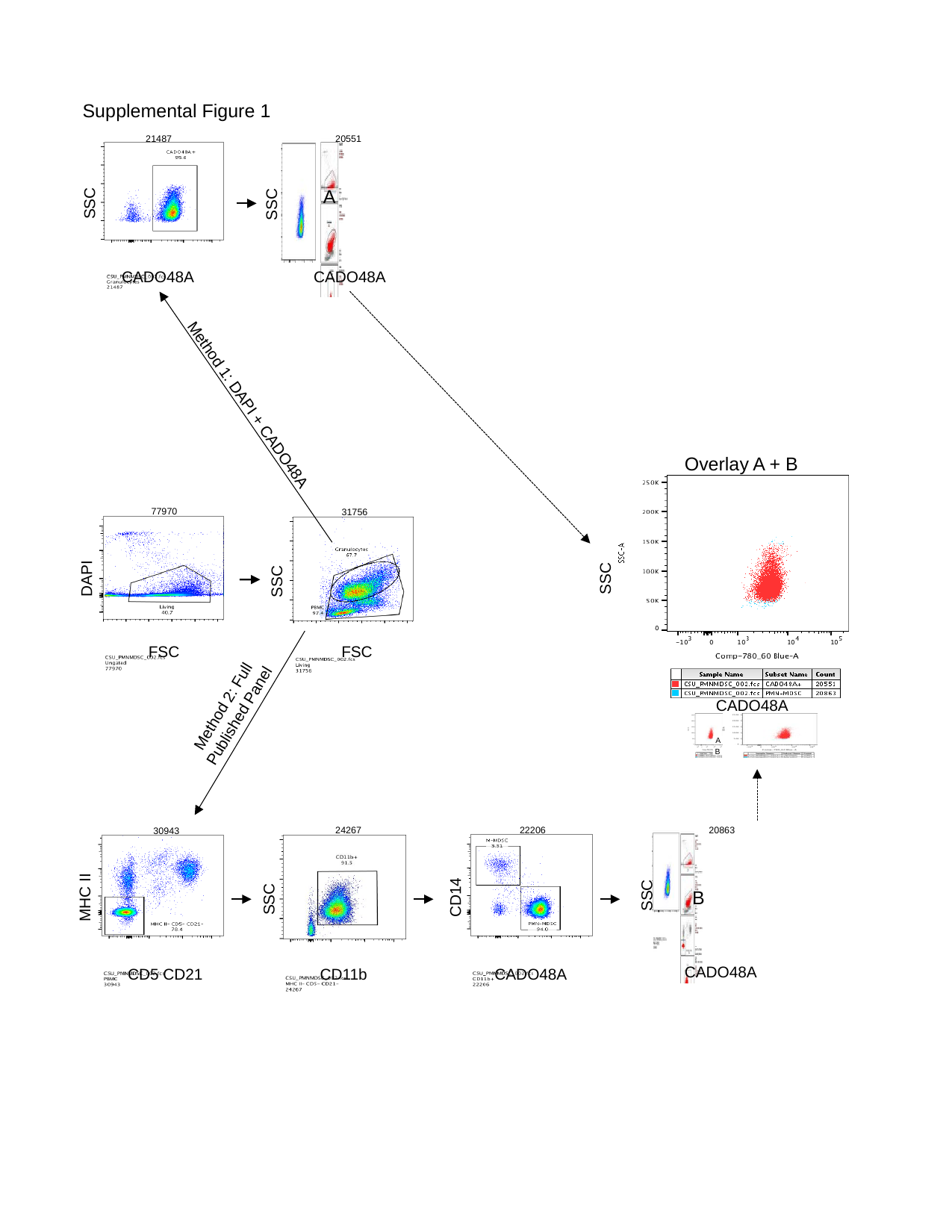

Supplemental Figure 1
21487
20551
A
SSC
SSC
CADO48A
CADO48A
Method 1: DAPI + CADO48A
Overlay A + B
77970
31756
DAPI
SSC
SSC
FSC
FSC
Method 2: Full Published Panel
CADO48A
A
B
24267
22206
20863
30943
B
SSC
MHC II
CD14
SSC
CADO48A
CD5 CD21
CD11b
CADO48A

## Slide 5
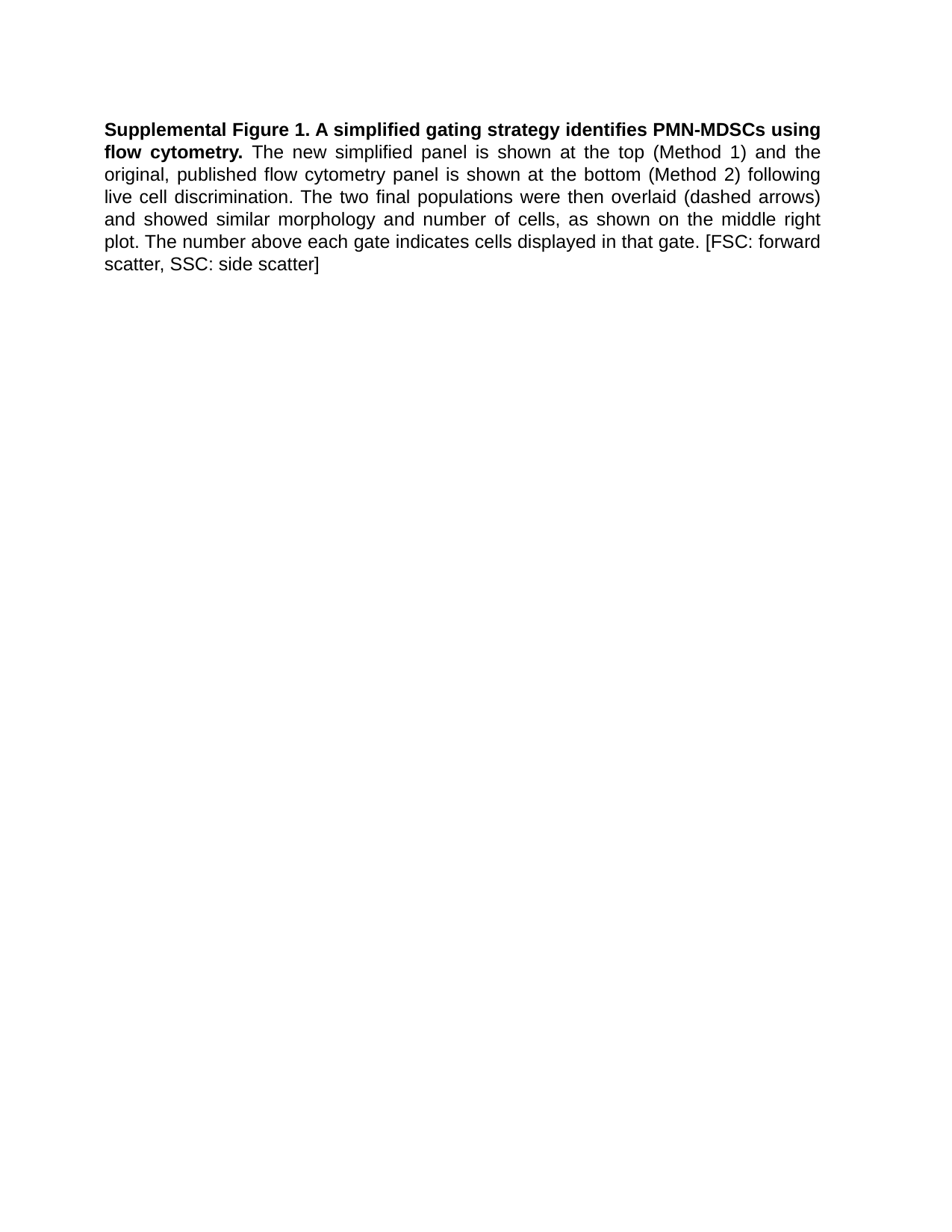

Supplemental Figure 1. A simplified gating strategy identifies PMN-MDSCs using flow cytometry. The new simplified panel is shown at the top (Method 1) and the original, published flow cytometry panel is shown at the bottom (Method 2) following live cell discrimination. The two final populations were then overlaid (dashed arrows) and showed similar morphology and number of cells, as shown on the middle right plot. The number above each gate indicates cells displayed in that gate. [FSC: forward scatter, SSC: side scatter]

## Slide 6
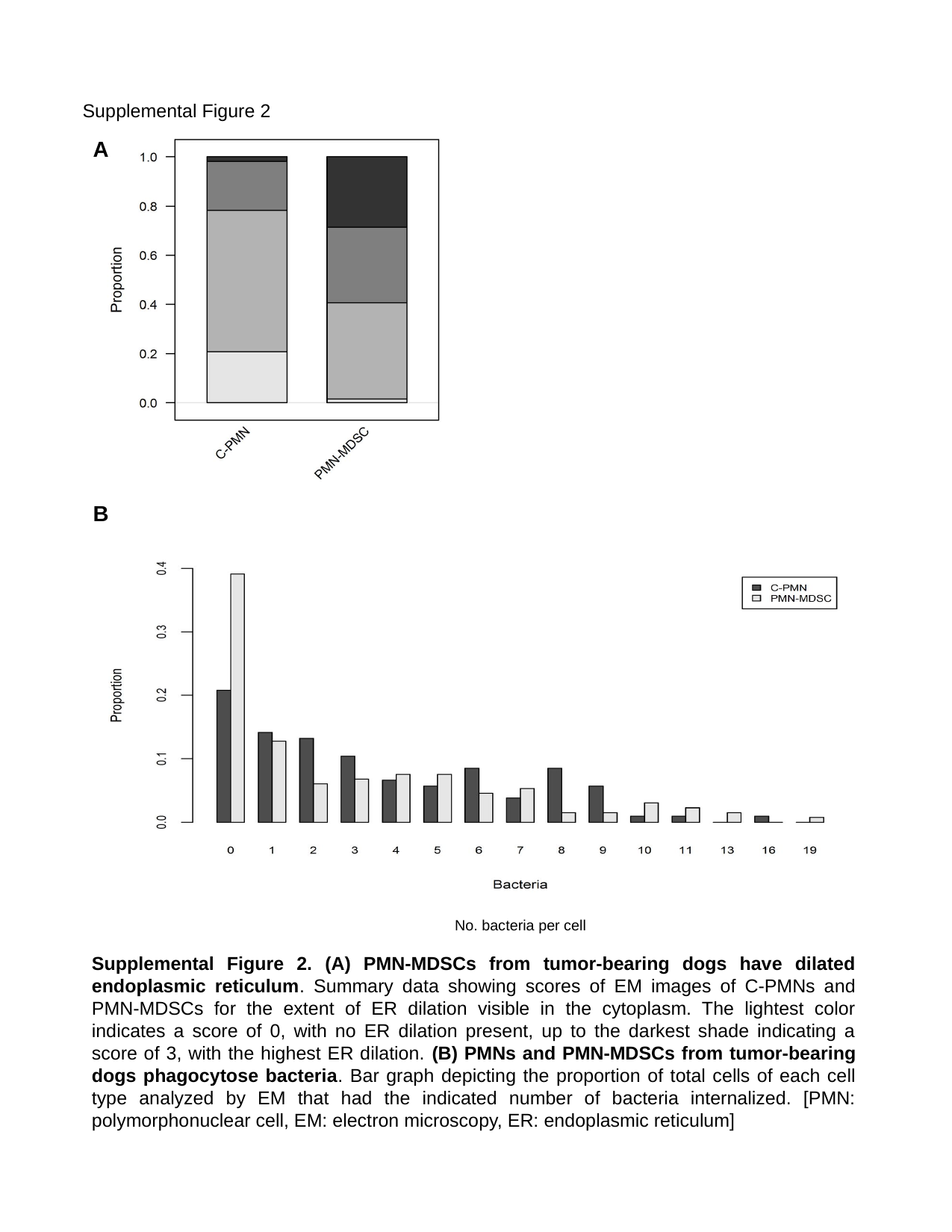

Supplemental Figure 2
A
B
No. bacteria per cell
Supplemental Figure 2. (A) PMN-MDSCs from tumor-bearing dogs have dilated endoplasmic reticulum. Summary data showing scores of EM images of C-PMNs and PMN-MDSCs for the extent of ER dilation visible in the cytoplasm. The lightest color indicates a score of 0, with no ER dilation present, up to the darkest shade indicating a score of 3, with the highest ER dilation. (B) PMNs and PMN-MDSCs from tumor-bearing dogs phagocytose bacteria. Bar graph depicting the proportion of total cells of each cell type analyzed by EM that had the indicated number of bacteria internalized. [PMN: polymorphonuclear cell, EM: electron microscopy, ER: endoplasmic reticulum]

## Slide 7
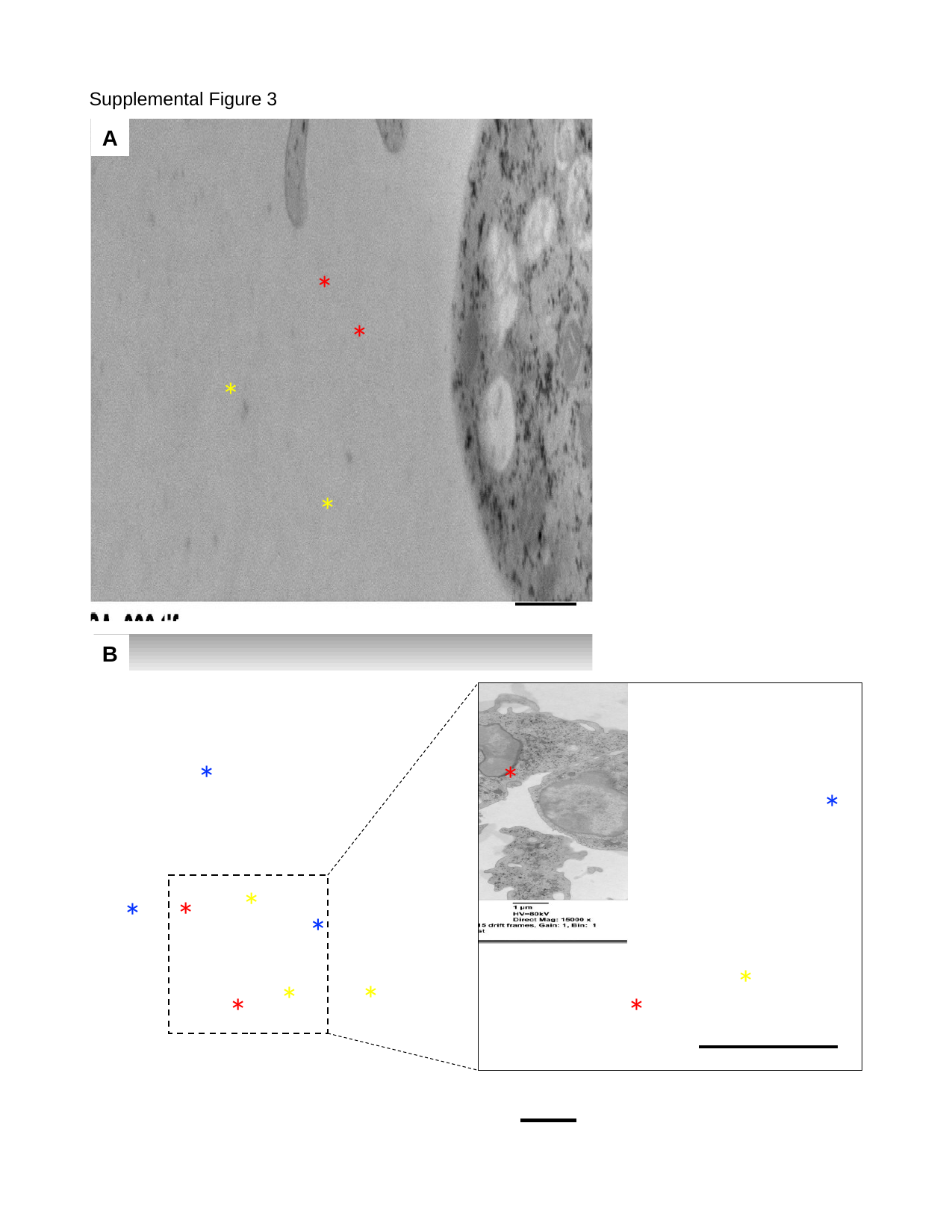

Supplemental Figure 3
A
*
*
*
*
B
*
*
*
*
*
*
*
*
*
*
*
*

## Slide 8
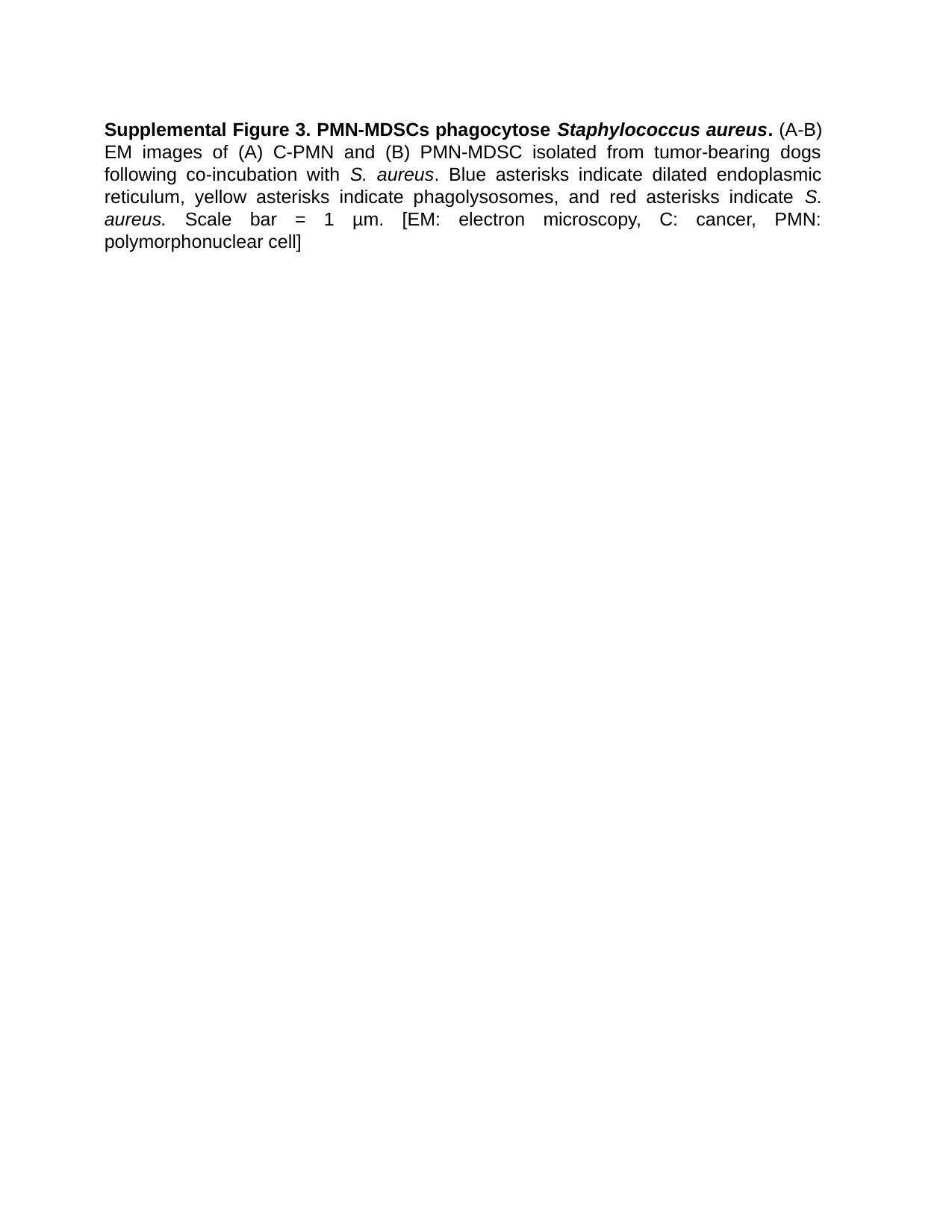

Supplemental Figure 3. PMN-MDSCs phagocytose Staphylococcus aureus. (A-B) EM images of (A) C-PMN and (B) PMN-MDSC isolated from tumor-bearing dogs following co-incubation with S. aureus. Blue asterisks indicate dilated endoplasmic reticulum, yellow asterisks indicate phagolysosomes, and red asterisks indicate S. aureus. Scale bar = 1 µm. [EM: electron microscopy, C: cancer, PMN: polymorphonuclear cell]
